# Screening of wild *cicer* germplasm from the fertile crescent against four pathotypes of *Ascochyta rabiei*

**DOI:** 10.1038/s41598-026-55103-w

**Published:** 2026-06-16

**Authors:** Emine Burcu Turgay, Merve Nur Ertaş Öz, Sibel Bülbül, Abdulkadir Aydoğan, Elif Atasayar, Havva Vildan Kılınç, Cuma Karaoğlu, Gözde Çelik Özer, Mine Ertem, Sirel Canpolat, Senem Tülek, Nilufer Akçi, Ersin Kavlak, Selin Gündüz

**Affiliations:** 1 Central Research Institute for Field Crops, Yenimahalle, Ankara, Türkiye; 2 Directorate of Plant Protection Central Research Institute, Yenimahalle, Ankara, Türkiye; 3 General Directorate of Plant Production, Ankara, Türkiye

**Keywords:** *Ascochyta rabiei*, *Wild relatives of chickpea*, *Ascochyta* blight, *Disease resistance*, Genetics, Plant sciences

## Abstract

*Ascochyta* blight is one of the most devastating diseases in chickpea-growing regions. Yield losses due to this disease can reach up to 100% in fields where susceptible genotypes are cultivated. To mitigate these impacts, resistant or tolerant varieties are recommended for effective disease control. However, *Ascochyta rabiei*, the causal pathogen, has the capacity to evolve into new pathotypes due to its genetic variability. Therefore, identifying novel sources of resistance is crucial for breeding programs. In Türkiye, four distinct pathotypes of *Ascochyta rabiei* are known, which differ in aggressiveness, with Pathotype IV being the most virulent. In this study, 159 wild accessions of *C. reticulatum* and *C. echinospermum*, together with seven *C. arietinum* genotypes comprising six registered cultivars and one desi-type genotype, were phenotypically screened against four *A. rabiei* pathotypes to evaluate their resistance responses. Statistical analysis revealed significant differences (p < 0.01) among genotypes in their reactions to the four pathotypes. Furthermore, cluster analysis identified promising genotypes that may serve as potential resistance sources. Among the clusters, C1 exhibited the lowest mean reaction scores, highlighting its promise for breeding purposes. Notably, genotype 592, a *C. reticulatum* accession collected from Savur, Mardin, demonstrated resistance to all pathotypes, making it a valuable candidate for further research. Future studies will focus on elucidating the genetic background of these promising genotypes to support their integration into breeding programs aimed at enhancing resistance against *Ascochyta* blight.

**Supplementary Information:**

The online version contains supplementary material available at https://doi.org/10.1038/s41598-026-55103-w.

## Introduction

Chickpea (*Cicer arietinum* L.) is a diploid (2n = 2 ×  = 16), self-pollinated pulse crop cultivated in more than 50 countries worldwide and represents one of the most important grain legumes for human and animal nutrition^1,2^. It also contributes to sustainable cropping systems through its role in crop rotation and biological nitrogen fixation^2^. Chickpea is currently the third most widely cultivated legume globally after common bean and pea^1^.

As one of the earliest domesticated grain legumes, chickpea is considered among the founder crops of Neolithic agriculture, as supported by archaeobotanical evidence from the Fertile Crescent^3,4^. Its primary center of origin is associated with southeastern Türkiye and neighboring regions of Syria, where its wild progenitor, *Cicer reticulatum*, occurs naturally^5,6^. Taxonomically, chickpea belongs to the family *Fabaceae*, subfamily *Faboideae*, and the genus *Cicer*. Most species within this genus are diploid, with 2n = 16 chromosomes^2^. The genus *Cicer* comprises approximately 43–44 species, including 9 annual and about 34–35 perennial species. Among these, *C. arietinum* is the only domesticated species cultivated worldwide, whereas the remaining species constitute valuable wild genetic resources for breeding programs (Smykal et al.^7,^^8^). Consistent with the importance of this region as a center of diversity, the flora of Türkiye includes 10 *Cicer* species, four of which are endemic: *C. echinospermum*, *C. floribundum*, *C. isauricum*, and *C. reticulatum*^9–11^.

Chickpea is one of the most important pulse crops in Türkiye. In 2023, chickpea production reached 580 thousand tons, with an average yield of 126 kg da⁻^1^ across a cultivation area of 4.6 million da^12^. Its importance in Türkiye is associated with its adaptation to dryland farming systems, economic value, and role in traditional diets. Under favorable conditions, chickpea can reach a yield potential of 3.5–5.0 t ha⁻^1^ and is generally regarded as a low-input pulse suited to dry and temperate environments^13^. However, actual yields remain constrained by both abiotic and biotic stress factors^14^.

Among biotic stress factors, *Ascochyta* blight, caused by the fungal pathogen *Ascochyta rabiei* (Pass.) Labr., is one of the most devastating diseases affecting chickpea cultivation both globally and in Türkiye. This pathogen severely reduces both yield and quality, with reported losses of up to 100% in susceptible varieties under favorable environmental conditions (Ilarsalan and Dolar^15–17^). Symptoms primarily manifest on the above-ground parts of the plant, leading to stem breakage and eventual plant death. The most distinctive symptom is the appearance of concentric rings with pycnidia formation, which are visible fungal fruiting bodies. The disease is transmitted through seeds, airborne spores, or water splash, with infections possible at any growth stage. *A. rabiei* thrives in cool, humid environments and can develop under temperatures ranging from 10 °C to 35 °C (Sharma and Gosh^18^; Manjunatha et al.^19^). One of the primary reasons for the high adaptability of *A. rabiei* is its capacity for sexual reproduction. The pathogen undergoes sexual reproduction on plant debris, facilitating the emergence of new variants through meiotic recombination. Due to its heterothallic nature, two distinct mating types (*Mat 1.1* and *Mat 1.2*) are required for sexual reproduction. *A. rabiei* exhibits both sexual and asexual reproductive cycles, where asexual reproduction primarily contributes to rapid disease spread, while sexual reproduction promotes genetic recombination and the emergence of pathotypes with varying levels of virulence^20,21^. During its sexual cycle, the pathogen forms perithecia that produce asci, each containing eight ascospores, which play a key role in generating genetic diversity (Trapero-Casas et al., 2012,Navas-Cortés et al.,^22^). *A. rabiei* is prevalent in most chickpea-growing regions worldwide, including Australia, where the pathogen can cause severe disease outbreaks. The distribution of mating types varies among regions, and the presence of both MAT1-1 and MAT1-2 idiomorphs has been reported in several pathogen populations (Navas-Cortés et al.^22,^^23^,Nalçacı et al.^24,^^25^).

*A. rabiei* has been classified into various race and pathotype groups by different researchers based on its pathogenicity and virulence characteristics. Studies have reported classifications ranging from 3 to 14 pathotypes or races, depending on the pathogen’s ability to infect chickpea genotypes^26–28^,Navas-Cortés et al.^22,^^29^. Reddy and Kabbabeh^27^ assessed the virulence of *A. rabiei* using six chickpea genotypes and identified six distinct pathotype groups. Similarly, Navas-Cortés et al.^22^ found six pathotype groups using a comparable set of genotypes. In the same year, Udupa et al.^30^ reported the presence of three pathotypes. Later, Chen et al.^20^ tested the isolates used by Reddy and Kabbabeh^27^ on the genotypes defined by Udupa et al.^30^ and discovered that five of the six previously identified races belonged to Pathotype I, while the sixth race was classified under Pathotype II. They further suggested that the three-pathotype system was simpler and more practical compared to other grouping methods. Following this, the three-pathotype model proposed by Udupa et al.^30^, which required only four genotypes for classification, became widely adopted.

Plant pathogenic fungi like *A. rabiei* can alter their genetic structures in response to the resistance levels of their hosts, a process explained by host–pathogen co-evolution^31^. Populations of *A. rabiei* are subjected to selective pressure by resistant chickpea genotypes developed through breeding programs (Nalçacı et al.^24^). This evolutionary pressure has led the pathogen to develop increasingly aggressive strains over time^21^, suggesting the potential emergence of new pathotypes with enhanced virulence in the future^32^. Until 2011, only three pathotypes were recognized globally. However, after 2011, new isolates classified as Pathotype IV were identified and shown to be more aggressive than previously recognized pathotypes^32^,Nalçacı et al.^33^). Currently, *A. rabiei* is divided into four pathotype groups based on virulence levels in four different chickpea genotypes in Türkiye. Before 2016, several resistant and intermediately resistant varieties were available to farmers. However, the emergence of the highly aggressive Pathotype IV during surveys conducted between 2014 and 2016 (Nalcaci et al.^33^) rendered most of these varieties moderately susceptible or fully susceptible. In chickpea-growing regions of Türkiye, Pathotype IV is now widely disseminated. Following the emergence of this new pathotype, the Turkish cultivars Gökçe, Azkan, and Çağtay, previously regarded as resistant or tolerant, are now classified as susceptible (Kabakçı and Özer^34,^^35^.

To effectively integrate critical information into breeding programs, it is essential to determine the levels of *Ascochyta* blight resistance in the national chickpea germplasm. A variety of disease management strategies are available to combat *Ascochyta* blight, including the breeding of resistant cultivars, chemical treatments, and cultural practices. Among these, developing resistant cultivars is considered the most promising, as it provides a long-term and eco-friendly solution for managing *Ascochyta* blight in the field. Many breeding programs prioritize this approach due to its sustainability and field applicability. However, while the breeding of resistant chickpea varieties addresses the problem temporarily, the emergence of more aggressive *Ascochyta* blight pathotypes often renders previously resistant varieties moderately to fully susceptible.

Domestication-related bottlenecks have strongly constrained the genetic diversity available in cultivated chickpea. Abbo et al.^31^ described four major evolutionary bottlenecks in cultivated chickpea: the restricted distribution of its wild progenitor *C. reticulatum*, the founder effect associated with domestication, the shift from autumn to spring sowing, and the replacement of traditional landraces by elite cultivars. These processes narrowed the genetic base of cultivated chickpea and increased the importance of wild relatives as sources of novel adaptive traits. Consistently, von Wettberg et al.^6^ showed that a large proportion of the genetic diversity present in *C. reticulatum* remains absent from modern breeding programs. The sister species *C. echinospermum* also represents a valuable genetic reservoir, although reproductive barriers may limit its direct use in some crosses^36^.

Nevertheless, exploiting this substantial genetic reservoir could be the key to enhancing chickpea resilience against *Ascochyta* blight. Prior to 2013, the global collection of wild *Cicer* species was relatively limited, comprising only 18 initial accessions of *C. reticulatum* and 10 accessions of *C. echinospermum*^37^. However, a collection expedition to the chickpea domestication region in Southeastern Türkiye in 2013 dramatically expanded the gene pool. This mission successfully gathered 371 accessions, sampling a vast range of environmental conditions from Adıyaman in the west to Şırnak in the east, Urfa in the south, and Bingöl in the north, covering diverse rainfall, temperature, altitude, and soil types^6^.

Following this collection expansion, the most aggressive *A. rabiei* population from Australia was tested against 197 wild chickpea species, including 149 *C. reticulatum* and 48 *C. echinospermum*, in a study conducted by Newman et al.^38^. The researchers identified several *C. reticulatum* genotypes with resistance to the pathogen, suggesting that these resistant sources could be utilized in breeding programs to develop new chickpea varieties with enhanced resilience to *Ascochyta* blight.

In summary, the domestication of chickpeas has led to a significant decline in genetic diversity, creating challenges for breeders. As a result, exploring germplasm collections is crucial for identifying new sources of resistance for breeding programs^39^. Türkiye, being the center of origin for chickpeas, holds great potential for uncovering novel genetic resources. However, the narrowing of the genetic base during breeding, coupled with the emergence of more virulent *A. rabiei* pathotypes, has rendered many developed varieties susceptible. This situation underscores the urgent need for new resistance sources in managing *Ascochyta* blight.

To address this need, the objectives of this study were to evaluate the resistance responses of 166 chickpea genotypes, primarily comprising 159 wild accessions of *C. reticulatum* and *C. echinospermum*, together with seven cultivated *C. arietinum* genotypes, against four Turkish pathotypes of *A. rabiei* under controlled greenhouse conditions; to identify genotypes with broad-spectrum resistance, particularly against the highly virulent Pathotype IV; and to characterize resistance patterns using statistical and multivariate approaches to support the use of promising wild germplasm in chickpea resistance breeding programs.

## Materials and methods

### plant materials

**Fig. 1:**
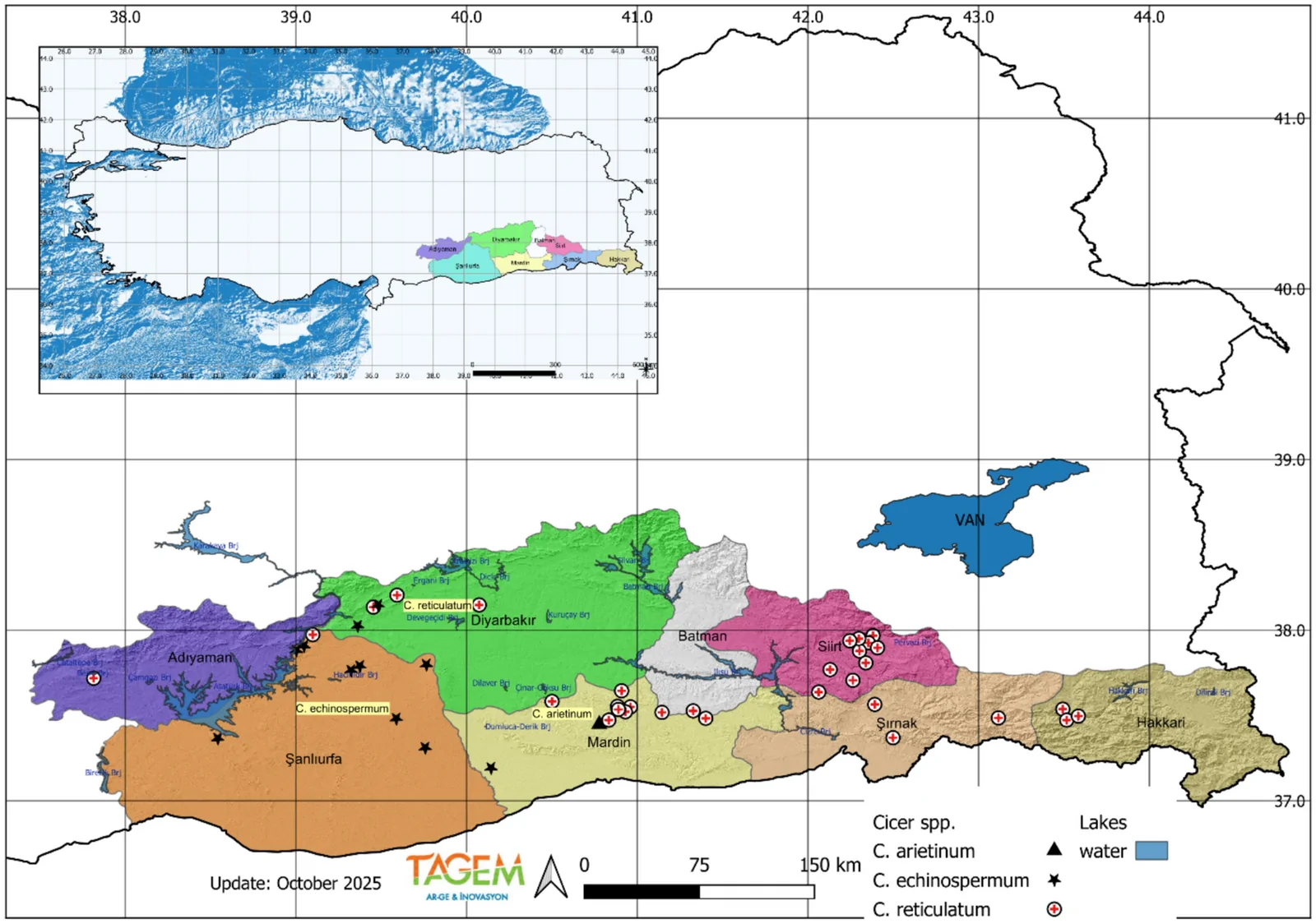
Geographic distribution of 166 chickpea germplasm accessions collected from the Fertile Crescent and southeastern and eastern regions of Türkiye. Different symbols represent species groups as shown on the map: *C. reticulatum* (** +**),* C. echinospermum* ( )*, and C. arietinum* ( ) Sampling locations span multiple provinces, reflecting the diversity of ecological conditions from which the germplasm was obtained. The map was created by the authors using QGIS software, version 3.30.2 (QGIS Development Team; https://qgis.org/), based on sampling coordinates and publicly available spatial datasets, including administrative boundary data for Türkiye and hillshade derived from the ASTER Global Digital Elevation Model. No copyrighted map was modified or reproduced.

The plant material used in this study comprised 159 wild chickpea accessions (*C. reticulatum* and *C. echinospermum*) and seven *C. arietinum* genotypes, including six registered cultivars widely grown in Türkiye and one desi-type genotype. All genotypes were obtained from the Türkiye Seed Gene Bank under official authorization. Exact collection site data (latitude and longitude) are not publicly available; however, all genotypes are preserved within the Gene Bank system, ensuring formal deposition and traceability. All necessary legal permissions for the use of the study material have been obtained from the Republic of Türkiye Ministry of Agriculture and Forestry Field Crops Central Research Institute Turkish Gene Bank. There is no objection to using the material for research purposes. Additional comprehensive information on *C. reticulatum* and *C. echinospermum* can be found in von Wettberg et al.^6^. Moreover, six registered *C. arietinum* cultivars commonly cultivated in Türkiye and one desi-type genotype were included in the study (Table 1). Regarding seed types, the collection included 7 Kabuli-type genotypes and 159 Desi-type genotypes. The genotypes acquired from the Türkiye Seed Gene Bank were originally collected from regions in Southeastern Türkiye and parts of the Fertile Crescent Table 2 (Fig. 1). Wild germplasm was specifically sourced from eight provinces (Adıyaman, Diyarbakır, Hakkari, Mardin, Siirt, Şanlıurfa, and Şırnak) across a total of 14 districts within these provinces (Fig. 1).

**Table 1:** Summary of chickpea germplasm used in the study, including species classification, number of accessions, and geographic origin in Türkiye.

Province	Species	Accessions (n)	Prefix	Accesion code number/Varieties
Adıyaman	*C. reticulatum*	4	Oyali	074,080, 082,112
	*C. echinospermum*	1	KoklK	038
Diyarbakır	*C. echinospermum*	1	Cermi	075
	*C. echinospermum*	3	Gunas	061,062, 100
	*C. reticulatum*	4	Dagar	22, 25, 26, 28
	*C. reticulatum*	3	Egil	063, 065, 071
	*C. reticulatum*	4	Kalka	061, 064, 065, 067
	*C. reticulatum*	5	Kesen	062, 073, 074, 075,101
Hakkâri	*C. reticulatum*	5	Kaval	001, 002, 003, 010, 013
	*C. reticulatum*	1	Olgun	026
	*C. reticulatum*	3	Ayval	001,005,006
	*C. reticulatum*	2	Uzumc	008, 012
Mardin	*C. reticulatum*	3	Yanıl	019, 021, 022
	***C. arietinum***	**1**	**Desi-crop**	**Desi-crop**
	*C. reticulatum*	1	Bari1	067
	*C. reticulatum*	3	Bari2	062, 063, 072
	*C. reticulatum*	3	Bari3	072C, 106D, 100
	*C. reticulatum*	5	Sarik	064, 065, 066, 067, 073
	*C. reticulatum*	5	Yolag	065, 066, 068, 069, 071
	*C. reticulatum*	2	Unknown	Unknown (2)
	*C. reticulatum*	3	Besev	081, Unknown (2)
	*C. reticulatum*	4	Derei	064, 070,071,Unknown (1)
	*C. reticulatum*	3	Kayat	064, 065, 077
	*C. reticulatum*	4	Hisar	017, 021, 025, 026
	*C. reticulatum*	3	Konak	046, 048, 050
	*C. reticulatum*	4	Pınar	044, 049, 056, 060
	*C. reticulatum*	4	Savur	063, 080, 033, 036
Siirt	*C. reticulatum*	1	Golko	001
	*C. reticulatum*	3	Dogan	026, 033, 038
	*C. reticulatum*	4	Ekind	045, 053, 059, 060
	*C. reticulatum*	1	Umurl	501
	*C. reticulatum*	2	Tasdi	025, 026
	*C. reticulatum*	2	Tuzca	027, 043
	*C. reticulatum*	1	Koprü	024
	*C. reticulatum*	4	Golge	032, 034, 036, 040
	*C. reticulatum*	1	Erenk	002
	*C. reticulatum*	2	Yanıl	012, 013
Şanlıurfa	*C. echinospermum*	6	Deste	063, 064, 073, 078, 079, 080
	*C. echinospermum*	3	Karab	066, 091B, 171
	*C. echinospermum*	1	Ortan	061
	*C. echinospermum*	5	S2Drd	061, 062, 065, 100, 105
	*C. echinospermum*	5	Erge	027, 028, 030, 033, 035
	*C. echinospermum*	4	Karpu	04, 010, 022, 026
	*C. echinospermum*	3	Kayal	028, 032, 038
	*C. echinospermum*	1	KayaP	36
	*C. echinospermum*	4	NarlıP	022, 024, 025, 027
	*C. echinospermum*	2	isoha	002, 042
	*C. echinospermum*	2	Otlu	005, 026
Şırnak	*C. reticulatum*	1	CudiB	022C
	*C. reticulatum*	1	CudiA	128
		4	Unknown	Unknown (4)
	*C. reticulatum*	8	Sirna	031,034,035, 036,043, 064, 070, 090
	*C. reticulatum*	5	Kayma	001, 034, 039, 046, 048
Total		160		
Chickpea varieties	***C. arietinum***	**6**	**-**	**Atabay, Azkan, Arda, Aksu,Hasanbey, Çakır**
Total		166		

**Note:** A total of seven *C. arietinum* genotypes were included in this study, comprising six registered cultivars (Atabay, Azkan, Arda, Aksu, Hasanbey, and Çakır) and one desi-type genotype (Desi-crop). These cultivated genotypes are highlighted in bold in the table for clarity.

**Table 2:** Identification numbers and corresponding accession codes of chickpea genotypes evaluated for resistance to *A. rabiei.*

Accession codes	ID NO	Accession codes	ID NO	Accession codes	ID NO	Accession codes	ID NO
Bari1_067	6	Savur_033	683	Dogan_033	493	Tuzca_027	691
Unknown	12	Karab_091B	232	Dogan_038	498	Tuzca_043	707
Unknown	13	Karab_171	238	Ekind_045	509	Yanıl_012	713
Bari2_062	15	Kayat_064	245	Ekind_053	517	Yanıl_013	714
Bari2_063	16	Kayat_065	246	Ekind_059	523	Yanıl_019	718
Bari2_072	21	Kayat_077	254	Ekind_060	524	Yanıl_021	719
Bari3_072C	32	Kesen_062	259	Golge_032	528	Yanıl_022	720
Bari3_100	41	Kesen_073	269	Golge_034	530	Desi_crop	721
Bari3_106D	47	Kesen_074	270	Golge_036	532	Dagar_22	806
Unknown	61	Kesen_075	271	Golge_040	536	Dagar_25	808
Unknown	68	Kesen_101	274	Erenk_002	543	Dagar_26	809
Besev_081	70	Ortan_061	320	Hisar_017	544	Dagar_28	811
Cermi_075	82	Oyali_074	335	Hisar_021	548	Erge_027	825
Unknown	91	Oyali_080	338	Hisar_025	552	Erge_028	826
Unknown	100	Oyali_082	340	Hisar_026	553	Erge_030	828
CudiB_022C	102	Oyali_112	358	Olgun_026	554	Erge_033	831
CudiA_128	116	Sarik_064	361	Kayma_001	557	Erge_035	833
Unknown	117	Sarik_065	362	Kayma_034	567	Karpu_04	921
Unknown	118	Sarik_066	363	Kayma_039	572	Karpu_010	925
Derei_064	147	Sarik_067	364	Kayma_046	579	Karpu_022	929
Unknown	148	Sarik_073	367	Kayma_048	581	Karpu_026	931
Derei_070	153	Savur_063	377	Umurl_501	583	Kayal_028	939
Derei_071	154	Savur_080	379	Konak_046	588	Kayal_032	943
Deste_063	163	Sirna_064	386	Konak_048	590	Kayal_038	949
Deste_064	164	Sirna_070	391	Konak_050	592	KayaP_36	958
Deste_073	170	Sirna_090	401	koprü_024	594	KoklK_038	991
Deste_078	175	S2Drd_061	408	Otlu_005	598	NarlıP_022	1063
Deste_079	176	S2Drd_062	409	Otlu_026	611	NarlıP_024	1065
Deste_080	177	S2Drd_065	412	Yolag_065	651	NarlıP_025	1066
Egil-_063	180	S2Drd_100	414	Yolag_066	652	NarlıP_027	1068
Egil-_065	182	S2Drd_105	419	Yolag_068	654	Ayval_001	1117
Egil-_071	187	isoha_002	424	Yolag_069	655	Ayval_005	1121
Gunas_061	194	isoha_042	448	Yolag_071	657	Ayval_006	1122
Gunas_062	195	sirna_031	463	Pınar_044	662	Kaval_001	1124
Gunas_100	197	sirna_034	466	Pınar_049	667	Kaval_002	1125
Kalka_061	199	sirna_035	467	Pınar_056	674	Kaval_003	1126
Kalka_064	202	sirna_036	468	Pınar_060	678	Kaval_010	1133
Kalka_065	203	sirna_043	475	Savur_036	686	Kaval_013	1136
Kalka_067	205	Golko_001	485	Tasdi_025	688	Uzumc_008	1147
Karab_066	219	Dogan_026	486	Tasdi_026	689	Uzumc_012	1151

Each accession code corresponds to a unique genotype used in the study. ID numbers were assigned for statistical analysis and data management.

### Fungal cultures

The study utilized representative isolates of *A. rabiei* corresponding to four distinct pathotypes (I, II, III, and IV), which are maintained in the culture collection of the Disease and Pest Resistance Unit at the Central Research Institute for Field Crops and are registered as AB-1, AB-2, AB-3, and AB-4, respectively. These isolates were kindly provided by Prof. Dr. Canan Can (Gaziantep University, Department of Biology) and have been previously characterized based on differential host reactions. Pathotype classification was carried out using the reference varieties *ILC482*, *ILC1929*, *ICC12004*, and *ILC3279*, which have been widely adopted for *A. rabiei* pathotype identification^30,32^,Nalçacı et al. ^33^). The differential reactions of these varieties to pathotypes are as follows: *ILC1929*: Susceptible to all pathotypes; *ILC482*: Resistant to Pathotype I, but susceptible to Pathotypes II, III, and IV; *ILC3279*: Resistant to Pathotypes I and II, but susceptible to Pathotypes III and IV; *ICC12004*: Resistant to Pathotypes I, II, and III, but susceptible to Pathotype IV^32^. These reference varieties were used as controls in all experimental assays to verify pathotype classification and resistance screening.

### Inoculum preparation

Pathotype I, II, III, and IV isolates of *A. rabiei* were cultured for 24 to 25 days at 22 °C with a 12-h light/12-h dark cycle on sterile Chickpea Seed Meal Dextrose Agar (composition: 40 g/L chickpea powder, 20 g/L agar, and 20 g/L glucose)^40^. To prepare the inoculum, 20 mL of sterile distilled water was added to *A. rabiei* cultures grown for two weeks on Petri plates, and the pycnidiospores were suspended to a concentration of 5 × 10^5^ spores/mL. Chickpea genotypes were then inoculated using a handheld spray pulverizer for uniform application.

### Greenhouse tests

The greenhouse experiments were conducted under fully controlled conditions at the Plant Disease and Pest Resistance Unit of the Field Crops Central Research Institute in Türkiye. Due to their hard seed coats, seeds of wild *Cicer* accessions were mechanically scarified prior to sowing. Following scarification, the seeds were surface-sterilized by immersion in 2% NaOCl solution (Product No. 239305, Sigma-Aldrich, USA) for three minutes, followed by three rinses with sterile distilled water for one minute each.

The sterilized seeds were sown in 9-cm-wide pots containing a peat:sand:perlite mixture at a 2:1:1 ratio. Six seeds were initially sown per pot to ensure adequate seedling emergence, particularly because wild *Cicer* accessions may show variable germination. After emergence, seedlings were thinned to three uniform plants per pot before inoculation. The pots were maintained under controlled greenhouse conditions at 22 °C, with a 12-h light/12-h dark photoperiod and 70%–80% relative humidity. Each genotype × pathotype combination was represented by three pots, each containing three seedlings, resulting in nine individual plants per combination.

The experimental layout followed a Randomized Complete Block Design (RCBD) as described by Newman et al.^38^. To promote successful infection, the pots were covered with plastic bags for 24 h immediately after inoculation and maintained under the same greenhouse conditions. Following this incubation period, the plastic covers were removed^20,28^,Nalçacı et al.^24^). Plants were watered as necessary.

The *A. rabiei* spore solution was sprayed onto 14-day-old seedlings of each genotype. No separate mock (non-inoculated) control treatment was included in this study, as the experimental design focused on comparative evaluation of genotype responses under uniform inoculation conditions. Instead, well-characterized differential chickpea genotypes (ILC1929, ILC482, ILC3279, and ICC12004) were included as reference controls to validate pathotype identity and disease reactions. Plants were visually monitored after inoculation to confirm symptom development and infection progress. Final disease severity scoring was performed two weeks post-inoculation using a 1–9 scale, as described by Singh and Reddy^41^ and Newman et al.^38^. This assessment time point was selected because clear and distinguishable disease symptoms had developed under the controlled greenhouse conditions, high inoculum pressure, and favorable humidity maintained in this study. Moreover, scoring all genotypes at the same seedling stage minimized potential confounding effects associated with severe tissue collapse or plant death at later stages, particularly under the more aggressive pathotypes.

### Assessment and statistical analysis

Disease severity assessments were performed based on the reaction groups defined in previous studies^17,27^. The reaction groups are classified as follows: I (Immune): 1,R (Resistant): 1.1–3; MR (Moderately Resistant): 3.1–5; S (Susceptible): 5.1–7; HS (Highly Susceptible): 7.1–9. To evaluate the differences in genotype reactions to the various pathotypes, two-way ANOVA (Analysis of Variance) and Tukey’s post-hoc tests were performed^38,42^. Additionally, hierarchical clustering using Ward’s method and Principal Component Analysis (PCA) were applied to group genotypes based on their reactions to pathotypes^38,42^. All statistical analyses were conducted using R Studio. Data manipulation was performed using the dplyr and tidyr packages. For the two-way ANOVA, a comprehensive suite of packages was utilized, including lme4^43^ for model fitting, emmeans^44^ for estimated marginal means, and multcomp^45^ alongside multcompView^46^ for post-hoc comparisons and compact letter displays. Additionally, pbkrtest^47^ was employed for statistical testing. To evaluate the relationship between wild relative species and clusters, a Chi-square test was conducted. Additionally, an independent two-sample t-test was performed for each pathotype to determine whether significant differences in disease severity exist between the species. For cluster analysis and Principal Component Analysis (PCA), the factoextra, FactoMineR, and cluster packages were used. All data visualizations were generated using the ggplot2 package. All statistical analyses and visualizations were conducted using R version 4.5.2.

## Results

### Pathotype reactions of chickpea genetic materials

A total of 166 chickpea genotypes comprising 7 *C. arietinum*, 37 *C. echinospermum*, and 122 *C. reticulatum* were evaluated against four distinct pathotypes of *Ascochyta* blight. The reference differential genotypes showed the expected disease reactions to the four A. rabiei pathotypes, confirming the reliability of the inoculation procedure and pathotype identity. ILC1929 was susceptible to all pathotypes, whereas ILC482 was resistant to Pathotype I and susceptible to Pathotypes II, III, and IV. ILC3279 was resistant to Pathotypes I and II but susceptible to Pathotypes III and IV, while ICC12004 was resistant to Pathotypes I, II, and III but susceptible to Pathotype IV. These reactions were consistent with previously reported differential responses and validated the pathotype classification used in this study.

The average disease severity for each pathotype are as follows: 2.72 for Pathotype I*,* 4.02 for Pathotype II, 5.06 for Pathotype III, and 6.90 for Pathotype IV, with median values of 2.6, 4.0, 5.3, and 7.3, respectively. Among the four, Pathotype IV exhibited the highest average disease severity, indicating its increased virulence. A closer examination of C1 revealed that 15 genotypes showed resistant to moderately resistant reactions to Pathotypes I, II, and III, whereas only genotype 592 was resistant to Pathotype IV. The two-way ANOVA revealed highly significant main effects (p < 0.001) for both chickpea genotypes and pathotypes, indicating substantial variation in inherent resistance and fungal virulence. Importantly, a significant genotype-pathotype interaction (p < 0.0001) was observed, demonstrating that the response of specific chickpea lines is dependent upon the pathotype encountered.

### Resistance group distribution according to pathotypes

The distribution of genotypes into resistance categories varied by pathotype: For Pathotype I, 72.8% of genotypes were classified as Resistant (R), whereas 28.3%, 14.4%, and 0.6% were classified as Resistant (R) for Pathotypes II, III, and IV, respectively. Furthermore, the percentages of genotypes classified as Moderately Resistant (MR) were: 23.4% for Pathotype I, 54.8% for Pathotype II, 33.7% for Pathotype III, 13.2% for Pathotype IV.

**Fig. 2:**
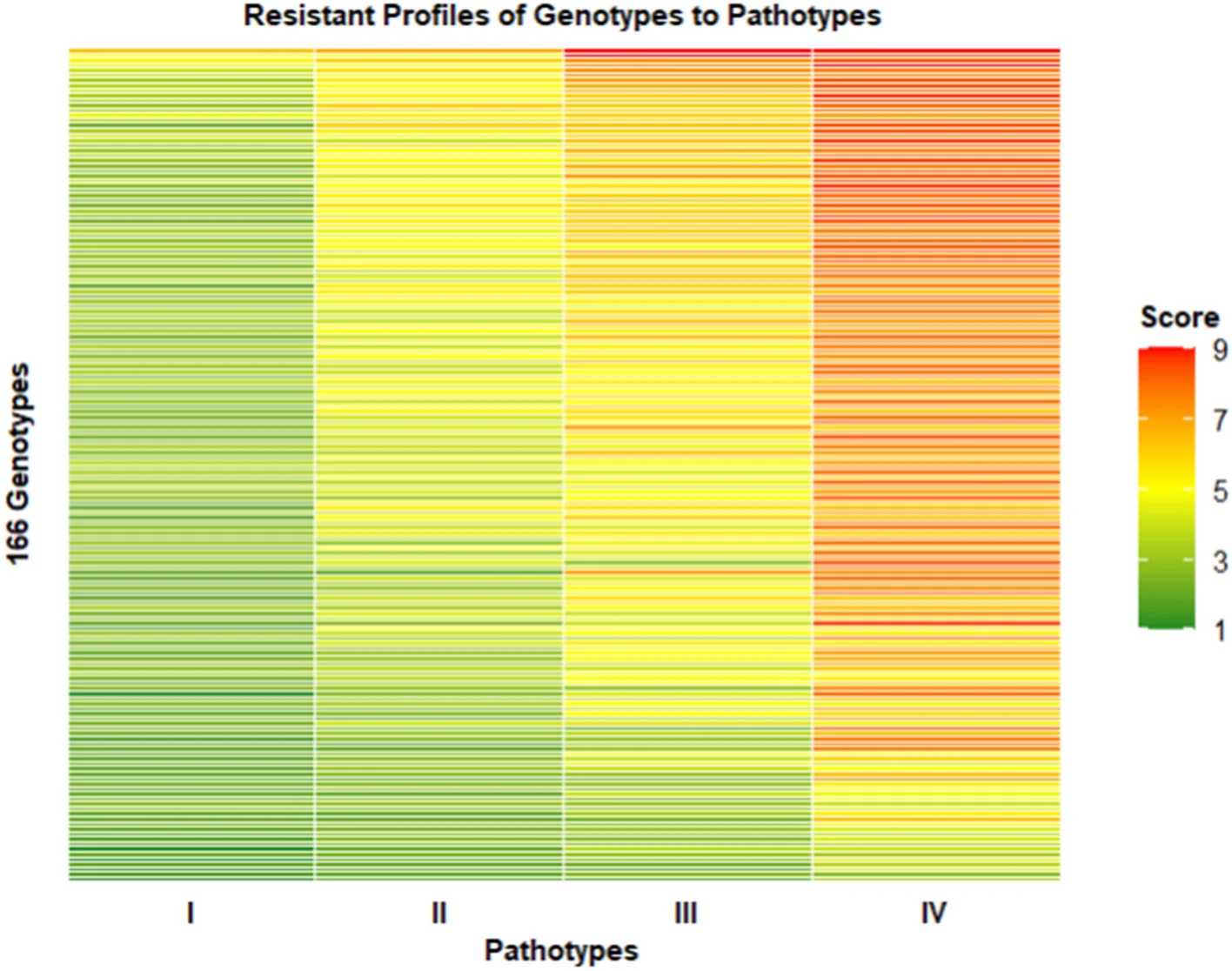
Heat map showing the resistance profiles of 166 chickpea genotypes against four *A. rabiei* pathotypes based on disease severity scores and post-hoc grouping.

Against the highly aggressive Pathotype IV, most genotypes were categorized as Susceptible (S) or Highly Susceptible (HS), whereas genotype 592 was the only genotype classified as Resistant. In addition, genotypes 180, 408, 412, and 338 exhibited immune reactions exclusively to Pathotype I. Several genotypes (6, 12, 13, 15, 16, 68, 176, 177, 180, 182, 408, 409, 412, 414, 424, 532, 543, 588, 590, 686, and 925) exhibited moderately resistant reactions across multiple pathotypes. These results were statistically supported by ANOVA and visualized through heatmap plot data (Table 3, Table 4; Fig. 2).

**Table 3:** Two-way analysis of variance (ANOVA) results showing the effects of genotype, pathotype, and their interaction on disease severity in chickpea genotypes.

Source of Variation	Sum Sq	Mean Sq	NumDF	DenDF	F Value	Pr(> F)
Genotype	1853,6	11,23	165	1326	13,4364	< 2.2e-16 ***
Pathotype	4789,6	1596,53	3	1326	1909,547	< 2.2e-16 ***
Genotype x Pathotype	901,8	1,82	495	1326	2,1791	< 2.2e-16 ***

Signif. codes: 0 ‘***’ 0.001 ‘**’ 0.01 ‘*’ 0.05 ‘.’ 0.1 ‘ ’ 1.

**Table 4:** Mean disease severity scores of chickpea genotypes in four hierarchical clusters across four *A. rabiei* pathotypes.

Clusters	Number of genotypes	Pat I	Pat II	Pat III	Pat IV
C1	25	1.97	2.53	3.18	4.39
C2	19	4.05	5.26	6.70	8.19
C3	43	2.43	3.13	4.27	7.15
C4	79	2.81	4.68	5.68	7.41

The first two principal components (Dim1 and Dim2) explain the majority of the variation in disease response. Genotype clustering reflects differences in resistance levels across pathotypes.

### Cluster analysis and pca results

**Fig. 3:**
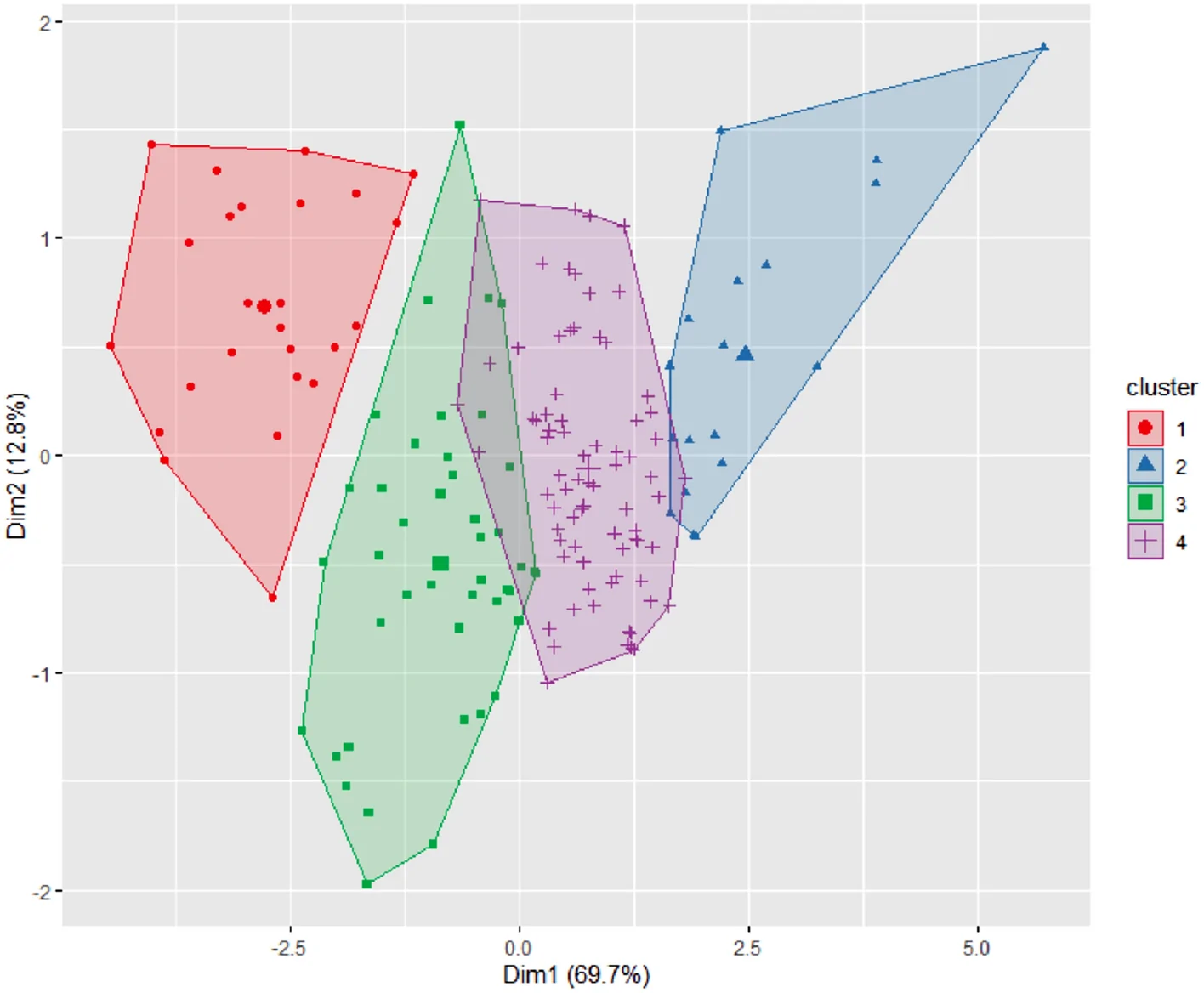
Principal component analysis (PCA) showing the distribution of chickpea genotypes based on disease severity responses to four *A. rabiei* pathotypes.

Cluster analysis identified four distinct clusters: C1, C2, C3, and C4. Among these, genotypes in Cluster C1 demonstrated the lowest average disease severity across all pathotypes. A closer examination of C1 revealed that 15 genotypes showed resistance to Pathotypes I, II, and III, whereas only genotype 592 was resistant to Pathotype IV. The Principal Component Analysis (PCA) revealed that Dim 1 and Dim 2 captured 82.5% of the total variation, indicating a high level of differentiation between pathotype virulence levels. This result is consistent with the separation of C1 and C2 in the PCA graph (Fig. 3). Notably, Cluster C2 contained genotypes with the highest average disease severity, predominantly classified as Susceptible (S) or Highly Susceptible (HS). Clusters C3 and C4 displayed mixed reactions: they were Resistant I or Moderately Resistant (MR) to Pathotype I, but their susceptibility increased as pathotype virulence intensified. These groups overlapped in the PCA plot, indicating some shared susceptibility patterns (Fig. 3; Table 4; Supplementary Materials).

To enhance the clarity and interpretability of the results, the distribution of all evaluated genotypes across resistance categories for each *A. rabiei* pathotype is summarized in Table 5. For each pathotype, all 166 genotypes were classified into one of five disease reaction groups (Immune, Resistant, Moderately Resistant, Susceptible, and Highly Susceptible); therefore, the total number of genotypes in each column equals 166.

**Table 5:** Number of chickpea genotypes in each resistance category across four *Ascochyta rabiei* pathotypes and their Post-hoc groups.

Resistant groups	PatI	Posthoc groups	PatII	Posthoc groups	PatIII	Posthoc groups	PatIV	Posthoc groups
Immune (I)	4	a	0	-	0	-	0	-
Resistant (R)	121	ab, abc, abcd, abcde	47	a, ab, abc	24	a, ab, abc, abcd, abcdefg, bcdefgh, cdefgh, efgh	1	a
Moderately Resistant (MR)	39	abcde, bcde, cde	91	abcd	56	abcde, abcdef, abcdefg	22	ab, abc, abcd, abcde, abcdef, abcdeg
Susceptible (S)	2	de,e	20	abcd, bcd, cd, d	52	abcdefgh, bcdefgh, cdefgh, defgh, efgh, fgh	80	abcdefgh, abcdefghi, bcdefghi, cdefghi
Highly Susceptible (HS)	0	-	8	de, e	34	gh,h	63	defghi, efghi, fghi, ghi, i
**Total (n)**	**166**		**166**		**166**		**166**	

The results clearly demonstrate a progressive shift in genotype distribution toward higher susceptibility levels with increasing pathotype virulence. While a large proportion of genotypes were classified as resistant or moderately resistant against Pathotype I, this proportion markedly decreased for Pathotypes II and III. In contrast, the majority of genotypes were classified as susceptible or highly susceptible against Pathotype IV. Notably, only a single genotype exhibited resistance to Pathotype IV, highlighting its high virulence and the limited availability of effective resistance sources within the evaluated germplasm.

The complete genotype-level classification and detailed resistance profiles are provided in the Supplementary Material (Table S1–S2) to allow comprehensive evaluation of individual genotype responses. The distribution of genotypes across disease reaction categories for each *A. rabiei* pathotype is provided in Supplementary Table S3.

### Pathotype reactions by species and region

**Fig. 4:**
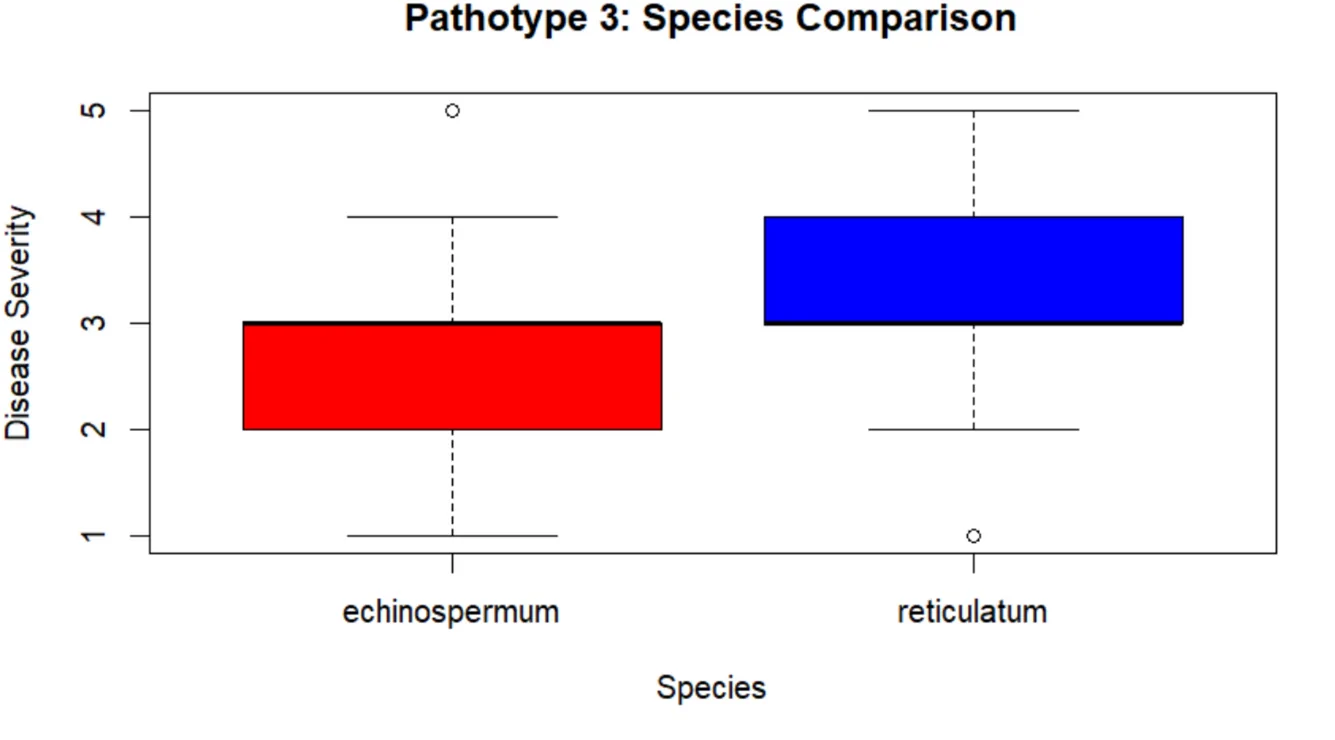
Comparison of *Ascochyta* blight disease severity between *C. reticulatum* and *C. echinospermum* in Pathotype III.

Among the 122 *C. reticulatum* genotypes tested, 74.5%, 26.2%, and 9.8% were resistant to Pathotypes I, II, and III, respectively. Only genotype 592 showed resistance to Pathotype IV. For the 37 *C. echinospermum* accessions: 75% were resistant to Pathotype I, 40.5% were resistant to Pathotype II, 32.4% were resistant to Pathotype III. However, no *C. echinospermum* genotype was resistant to Pathotype IV. Overall, *C. echinospermum* exhibited a higher resistance rate compared to *C. reticulatum*. Furthermore, the Chi-square test indicated a highly significant association between species and clusters (X^2^ = 29.904, df = 3, p < 0.001). In addition, independent two-sample t-test analyses performed for each pathotype revealed that *C. echinospermum* exhibited significantly lower mean disease severity compared to *C.* reticulatum (p < 0.05) (Fig. 4).

When the resistance status of wild chickpea species was evaluated based on regions, *C. reticulatum* genotypes demonstrating R or MR reactions to Pathotype IV were primarily collected from Mardin (Midyat, Savur district), Diyarbakır (Eğil district), and Siirt (Eruh district). For *C. echinospermum*, no resistant genotype was found; however, genotypes with MR reactions were identified in Şanlıurfa (Siverek, Viranşehir districts) (Fig. 1*, *Table 1).

## Discussion

*Ascochyta* blight is a widespread and devastating disease affecting chickpea cultivation both in Türkiye and globally. The pathogen, *A. rabiei*, exhibits a high capacity for virulence variation, which, combined with the limited genetic diversity in chickpea germplasm, poses significant challenges for breeding resistant varieties. This study is therefore crucial for advancing chickpea resistance breeding by identifying promising genotypes with enhanced resistance.

The cluster analysis conducted in this study highlighted Cluster 1 (C1) as the most significant group for future breeding efforts. The genotypes within C1 demonstrated strong resistance to multiple pathotypes, including Pathotype IV, the most virulent strain currently identified. The distribution of species within the four clusters was analyzed using the Chi-square independence test. The results revealed a highly significant relationship between species and cluster membership (X^2^ = 29.904, df = 3, p < 0.001). Two t-test statistical comparisons revealed that *C. echinospermum* accessions exhibited significantly higher resistance levels to Pathotype III compared to *C. reticulatum* (p < 0.05). However, no significant interspecific differences were detected for the other three pathotypes. This suggests that *C. echinospermum* may harbor resistance factors that are particularly effective against Pathotype III. A similar pattern was also reported by Newman et al.^38^.

Research on this topic in Türkiye has been relatively limited. A recent study by Lakmes et al.^48^ evaluated 305 interspecific hybrid lines derived from *C. reticulatum* and *C. echinospermum* under natural field conditions against races presumed to be Pathotype I and/or Pathotype II. The hybrids demonstrated significantly higher resistance compared to the crossbred cultivars. However, the current study is unique as it screened a broad wild chickpea genotype collection against Pathotype IV under controlled greenhouse conditions for the first time in Türkiye. This led to the identification of genotypes with notable resistance to this highly aggressive strain. Interestingly, in a similar study conducted in Syria, wild chickpea accessions were tested against Pathotype IV, yet none showed resistance^32^. This indicates a significant advancement in identifying resilient genotypes within Turkish germplasm.

Globally, several studies have focused on wild chickpea species for disease resistance. Pande et al.^49^ screened a population of 148 accessions representing seven wild species (*C. bijugum*, *C. cuneatum*, *C. echinospermum*, *C. judaicum*, *C. pinnatifidum*, *C. reticulatum*, and *C. yamashitae*) against a virulent *A. rabiei* isolate. Remarkably, a few accessions of *C. bijugum* and *C. judaicum* displayed complete resistance, with no visible lesions, while some lines of *C. pinnatifidum* and *C. reticulatum* exhibited low disease scores, classified as Resistant (R) or Moderately Resistant (MR). *C. bijugum* was particularly noteworthy, as certain accessions displayed 100% resistance across all tested plants.

Despite their promising resistance, *C. bijugum* and *C. judaicum* were excluded from breeding programs due to difficulties in achieving successful hybridization. In contrast, *C. echinospermum* and *C. reticulatum* showed compatibility with cultivated chickpeas, making them viable candidates for breeding programs aimed at enhancing resistance to *Ascochyta* blight. This compatibility underscores the importance of focusing on these species for integrating novel resistance genes into chickpea breeding lines.

Chickpea wild relatives have demonstrated resistance not only to *Ascochyta* blight but also to various other chickpea diseases. For instance, Kaur et al.^50^ tested multiple wild chickpea accessions for resistance against both *Ascochyta* blight and Botrytis gray mold, discovering that accessions from some of the seven species examined displayed significant resistance. Similarly, Knights et al.^51^ reported that *C. echinospermum* accessions showed notable resistance to Phytophthora root rot (*Phytophthora medicaginis*), highlighting its potential for breeding programs targeting multiple disease resistances.

Significant breeding research has also been conducted in Australia, where chickpea is an economically important pulse crop. Danehloueipour et al.^52^ performed a diallel mating design involving three *C. reticulatum* and one *C. echinospermum* accessions crossed with seven chickpea varieties. These crosses were tested under field conditions against a mixed field isolate. Diallel analysis revealed significant additive and dominant gene effects on disease resistance. The study demonstrated that resistance is controlled by both major and minor genes, suggesting polygenic inheritance.

In 2021, a comprehensive study was carried out with wild chickpea accessions collected from Türkiye, assessing both phenotypic and genotypic resistance. The study found that both *C. reticulatum* and *C. echinospermum* species exhibited high resistance levels, and specific SNP regions linked to this resistance were identified^38^. Notably, the study also discovered a correlation between the geographic regions where resistant *C. echinospermum* accessions were collected and their resistance levels.

Furthermore, six common accessions (Deste_064, S2Drd_061, Deste_079, Gunas_062, Gunas_100, and Kesen_101) were evaluated in both the Australian and Turkish studies. These genotypes were predominantly found to be resistant when tested against Australian isolates and showed R (Resistant) and/or MR (Moderately Resistant) reactions to Pathotypes I, II, and III from Turkish isolates. However, with the exception of *Deste_079* and *S2Drd_061*, these genotypes displayed MR reactions to Pathotype IV, while the other four (Deste_064, Gunas_062, Gunas_100, and Kesen_101) were classified as Susceptible (S) or Highly Susceptible (HS). This suggests that these accessions may have limited value as resistance sources against the highly virulent Pathotype IV under Turkish conditions, although they may still be useful for other breeding objectives or against less aggressive pathotypes.

Although some of the genotypes tested in this study overlap with those used by Newman et al.^38^, the key distinction between the two studies lies in the differences in isolate virulence. The fact that genotypes like Deste_064, Gunas_062, Gunas_100, and Kesen_101*,* which were found to be resistant in the Australian study, exhibited susceptible or highly susceptible reactions in the Turkish trials, provides strong evidence for pathotype-specific differences in virulence.

When examining the resistance breeding efforts for chickpea over the past two decades (Ertas Oz et al. 2023; Mart et al.^53,^^32^), the findings of the present study are particularly valuable. To date, there has been no prior study in Türkiye utilizing such a large wild population or screening against Pathotype IV the most virulent strain identified. This highlights the novelty and significance of the current findings.

One of the main limitations of this study is that the findings are based solely on experiments conducted under controlled greenhouse conditions. In addition, although final disease scoring was performed two weeks post-inoculation, this time point provided clear differentiation among genotypes and pathotypes under the controlled conditions used in this study. Nevertheless, longer-term and field-based validation will be important to confirm the stability of these resistance responses. Although greenhouse experiments provide a standardized and reproducible environment for evaluating host–pathogen interactions, they do not fully capture the environmental variability present under field conditions, such as climatic fluctuations, soil heterogeneity, and natural infection pressure. Therefore, the validation of the identified resistant genotypes under field conditions is essential for the accurate interpretation of the results and their effective integration into breeding programs. Future studies should focus on multi-environment field trials to confirm the stability and applicability of these resistance sources.

The genotypes identified as resistant or tolerant in this study represent a valuable resource for breeding programs aimed at developing *Ascochyta* blight-resistant chickpea varieties. These genotypes may contribute to the development of resilient cultivars adapted to *Ascochyta* blight-endemic regions, thereby supporting sustainable chickpea production in Türkiye and beyond.

## Supplementary Information

## Data Availability

Data will be made available on request from corresponding author.
