# Supplementary material for: Screening of wild cicer germplasm from the fertile crescent against four pathotypes of Ascochyta rabiei
Source: Sci Rep. 2026 Jun 16;16:27623. doi: 10.1038/s41598-026-55103-w (PMC13538585; doi:10.1038/s41598-026-55103-w)
Supplement: Supplementary file 1 [file 41598_2026_55103_MOESM2_ESM.docx]

**Supplementary Materials**: **Detailed Resistance Classification of Chickpea Genotypes Across *Ascochyta rabiei* Pathotypes**

**Table S1.** List of genotypes based on resistance groups across pathotypes

| I | Pat I | | | | Pat II | | | |
| --- | --- | --- | --- | --- | --- | --- | --- | --- |
|  | C1 | C2 | C3 | C4 | C1 | C2 | C3 | C4 |
|  | 180,408,412 |  | 338 |  |  |  |  |  |
|  | Pat III | | | | Pat IV | | | |
|  | C1 | C2 | C3 | C4 | C1 | C2 | C3 | C4 |
|  |  |  |  |  |  |  |  |  |
| R | Pat I | | | | Pat II | | | |
|  | C1 | C2 | C3 | C4 | C1 | C2 | C3 | C4 |
|  | 6,12,13,15,16,68,154,163,176,177,182,409,414,419,424,532,543,588,592,686,806,925 |  | 82,153,164,170,175,187,197,199,205,219,245,246,254,259,270, 271,274,340,364,367,498,509,528,530,536,583,598,611,651,652,654,688,689,721,808,809,811,921,929,949 | 21,41,47,61,91,100,102,116,118,147,148,202,203,232,238,320,358,361,362,363,379,386,391,401,448,485,486,493,517,523,548,554,557,567,579,581,594,657,662,674,678,683,691,713,720,825,828,831,931,939,1066,1117,1121,1122,1124,1125,1133,1151,At | 6,12,13,15,16,68,154,163,176,177,180,182,408,409,412,414,419,532,543,592,686,806,925 |  | 153,164,170,175,245,246,254,259,274,338,340,364,367,498,509,583,598,611,651,652,688,808,809,921 |  |
|  | Pat III | | | | Pat IV | | | |
|  | C1 | C2 | C3 | C4 | C1 | C2 | C3 | C4 |
|  | 6,12,13,15,16,154,163,176,177,408,409,412,414,419,543,592,925 |  | 164,274,367,583,598,611,654 |  | 592 |  |  |  |
| MR | Pat I | | | | Pat II | | | |
|  | C1 | C2 | C3 | C4 | C1 | C2 | C3 | C4 |
|  |  | 32,117,463,466,467,468,667,707,714,719,958,1065,1136,1147,Az,Ar,Ca | 590, 833 | 70,194,195,269,335,377,475,524,544,552,553,572,655,718,826,943,991,1063,1068,1126 | 424, 588 | 463,467,714,958,1065,1136,1147,Az,Ar,Ca | 82,187,197,199,205,219,270,271,528,530,536,590,654,689,721,811,833,929,949 | 21,91,100,147,148,194,195,232,238,269,320,335,358,362,363,377,379,386,391,448,475,486,493,517,523,524,544,552,553,554,567,572,579,581,594,655,662,674,678,713,718,825,826,828,831,931,939,943,991,1063,1066,1068,1117,1121,1122,1124,1125,1126,1133,1151 |
|  | Pat III | | | | Pat IV | | | |
|  | C1 | C2 | C3 | C4 | C1 | C2 | C3 | C4 |
|  | 68,180,182,424,532,588,686,806 | 1065 | 82,153,170,175,187,197,205,219,245,246,254,259,270,271,338,340,364,528,530,536,590,651,652,688,689,808,809,811,833,921,929,949 | 194,195,232,269,320,377,594,655,662,825,826,831,931,991,1063 | 6,12,13,15,16,68,176,177,180,182,408,409,412,414,419,424,532,543,588,686,925 |  | 590 |  |

**Table S2.** Continued list of genotype classifications

| S | Pat I | | | | Pat II | | | |
| --- | --- | --- | --- | --- | --- | --- | --- | --- |
|  | C1 | C2 | C3 | C4 | C1 | C2 | C3 | C4 |
|  |  | Ak, Ha |  |  |  | 32,117,466,468,667,707,719,Ak,Ha |  | 41,47,61,70,102,116,118,202,203,361,401,485,548,557,657,683,691,720,At |
|  | Pat III | | | | Pat IV | | | |
|  | C1 | C2 | C3 | C4 | C1 | C2 | C3 | C4 |
|  |  | 32,117,463,468,667,707,714,719,958,1136,1147,Ar | 199,498,509,721 | 21,41,47,61,70,91,100,102,116,118,147,148,202,203,238,335,358,361,362,363,379,386,391,401,448,475,485,486,493,517,523,524,544,548,552,553,554,557,567,572,579,581,657,674,678,683,691,713,718,720,828,939,943,1066,1068,1117,1121,1122,1124,1125,1126,1133,1151,At | 154,163,806 | 32 | 153,164,170,175,187,199,219,245,246,254,259,509,688,721,809,833,921,929,949 | 21,41,47,61,70,147,148,232,448,475,485,524,572,581,718,825,931,943,1063,1066,1068,1117,1121,1122,1151 |
| HS | Pat I | | | | Pat II | | | |
|  | C1 | C2 | C3 | C4 | C1 | C2 | C3 | C4 |
|  |  |  |  |  |  |  |  |  |
|  | Pat III | | | | Pat IV | | | |
|  | C1 | C2 | C3 | C4 | C1 | C2 | C3 | C4 |
|  |  | 466,467,Az,Ak,Ha,Ca |  |  |  | 117,463,466,467,468,667,707,714,719,958,1065,1136,1147,Az,Ar,Ak,Ha,Ca | 82,197,205,270,271,274,338,340,364,367,498,528,530,536,583,598,611,651,652,654,689,808,811 | 91,100,102,116,118,194,195,202,203,238,269,320,335,358,361,362,363,377,379,386,391,401,486,493,517,523,544,548,552,553,554,557,567,579,594,655,657,662,674,678,683,691,713,720,826,828,831,939,991,1124,1125,1126,1133,At |
